# Mechanically Stable Kondo Resonance in an Organic Radical Molecular Junction

**DOI:** 10.1021/acs.jpcc.4c05860

**Published:** 2025-01-30

**Authors:** Tristan Bras, Chunwei Hsu, Thomas Y. Baum, David Vogel, Marcel Mayor, Herre S. J. van der Zant

**Affiliations:** † Kavli Institute of Nanoscience, Delft University of Technology, Lorentzweg 1, Delft 2628 CJ, The Netherlands; ‡ Department of Chemistry, University of Basel, St. Johanns-Ring 19, 4056 Basel, Switzerland

## Abstract

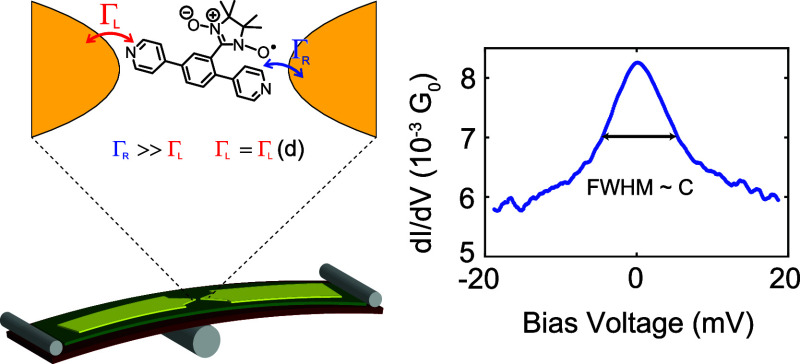

Organic radicals are promising candidates for molecular spintronics due to their intrinsic magnetic moment, their low spin–orbit coupling, and their weak hyperfine interactions. Using a mechanically controlled break junction setup at both room and low temperatures (6 K), we analyze the difference in charge transport between two nitronyl nitroxide radicals (**NNR**): one with a backbone in the *para* configuration, the other with a backbone in the *meta* configuration. We find that *para***-NNR** displays a Kondo resonance at 6 K, while *meta***-NNR** does not. Additionally, the observed Kondo peak in the differential conductance has a roughly constant width independent of the conductance, consistent with a scenario where the molecule is coupled asymmetrically to the electrodes.

## Introduction

A major objective of the field of molecular spintronics is to use the spin property of magnetic molecules as a platform to implement logic, memory, and sensing capabilities in electronics. Particularly, organic magnetic molecules stand out as an excellent candidate for spintronics applications as they offer several advantages over the currently used inorganic materials, including long spin decoherence times, a weak hyperfine interaction, and their naturally small size.^1−3^ Of special interest are radicals, molecules with an open shell called the singly occupied molecular orbital (SOMO). A half-filled orbital contributes one spin: a single radical thus has a total spin of 1/2, whereas a diradical can have a total spin of 0 (when the two spins are antiferromagnetically coupled) or 1 (when the two spins are ferromagnetically coupled). Radicals can carry a net charge, or they can be neutral. Being able to control the spin degree of freedom in single molecules allows us to study spin transport phenomena at the molecular level and could offer new functionalities as a result of built-in molecular properties. For example, recent studies have found radicals to display promising thermoelectricity properties,^4,5^ rectifying behavior,^6^ and to function as a molecular wire where the conductance increases with length.^7,8^

Experiments on molecular junctions with organic radicals display Kondo physics at cryogenic temperatures.^9−11^ Observation of a Kondo resonance in a molecular junction confirms the presence as well as the radical character of the molecule in the junction. So far, research has focused on the observation and manipulation of the Kondo resonance^10,11^ and, more recently, other magnetic effects such as magnetoresistance.^12,13^ The configuration of the backbone of the molecule is known to affect the molecular conductance: a *para* configuration generally has a higher conductance than a *meta* configuration due to quantum interference.^14,15^ The effect quantum interference in the molecular orbitals has on spin-related phenomena, such as the Kondo effect, is unknown. Here, we study two all-organic single radicals in a mechanically controlled break junction (MCBJ) setup at both room and cryogenic temperatures. We find that the configuration of the molecular backbone affects the coupling of the radical to the electrode.

## Methods

### Synthesis

**Figure 1:**
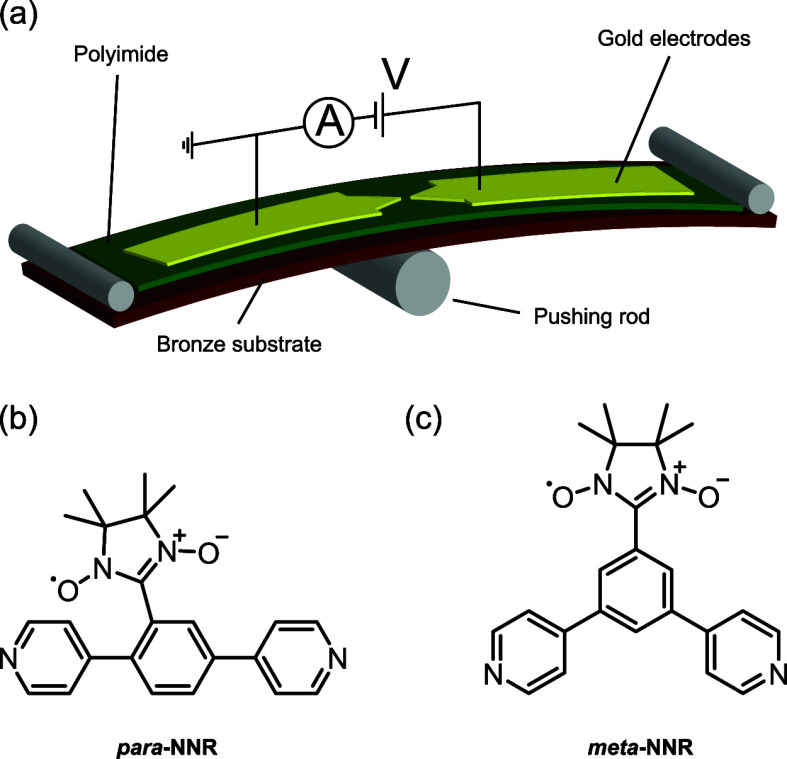
(a) Schematic of the two-terminal MCBJ setup. (b) Chemical structure of the *para*-nitronyl nitroxide radical. (c) Chemical structure of the *meta*-nitronyl nitroxide radical.

The two nitronyl nitroxide radicals *para***-NNR** and *meta***-NNR** both consist of three interlinked aromatic subunits as a backbone with the nitronyl nitroxide radical attached to the central benzene. Terminal pyridyl subunits act with their coordinating nitrogen as anchor groups. The difference between both model compounds is the substitution pattern of the central benzene ring, also giving the trivial names used in the manuscript. The two 4-pyridyl subunits are attached at the central benzene in the *para*-position with respect to each other in *para***-NNR**, favoring the communication through the backbone. As a consequence, the nitronyl nitroxide radical subunit is mounted asymmetrically with respect to the backbone’s symmetry: the radical couples in *ortho*-position with respect to one pyridyl group (the one on the left side in Figure 1b) which is expected to result in a reasonable communication between both subunits; the coupling to the other one is in the less favored *meta*-position. The *meta***-NNR** model compound has a higher symmetry. The 1,3,5-substitution pattern of the central benzene ring puts all three substituents in the *meta*-positions with respect to each other. The higher symmetry comes, however, at the price of a less efficient electronic communication between all three subunits.

**Figure 2:**
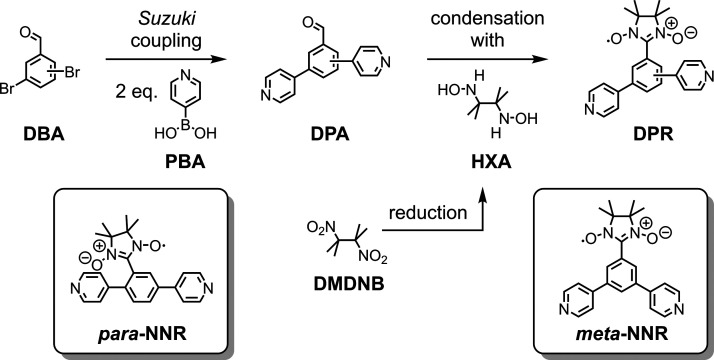
Three reaction steps making both structures of interest, *para***-NNR** and *meta***-NNR**, available. Synthetic protocols of *para***-NNR** are reported in ref (16), and the ones of *meta***-NNR** are available in the Supporting Information.

Both investigated model compounds, *para***-NNR** and *meta***-NNR**, were synthesized by a similar sequence of three reaction steps as displayed in Figure 2. Starting with the dibromobenzaldehyde (**DBA**) with the corresponding substitution pattern, both bromine atoms were substituted with 4-pyridyl groups by Suzuki coupling with 4-pyridine boronic acid (**PBA**), resulting in the di-4-pyridylbenzaldehyde (**DPA**). Condensation of the benzaldehyde with hydroxylamine (**HXA**) yields the desired dipyridyl-decorated nitronyl nitroxide radical (**DPR**). The hydroxylamine (**HXA**) was obtained by the reduction of 2,3-dimethyl-2,3-dinitrobutane (**DMDNB**). While the synthesis of *para***-NNR** has already been reported,^16^ the synthesis and characterization of *meta***-NNR** is available in the Supporting Information.

The conductance of the two molecules was characterized in an MCBJ setup (Figure 1a), at both room temperature and ∼6 K. A gold nanowire was broken by slowly bending the bronze substrate with the pushing rod. Eventually, the wire ruptured, and a molecular junction was formed. More details on the MCBJ method can be found in prior work.^17^ In short, before dropcasting the solution containing the molecule on the break junction, reference measurements were taken on the bare gold junctions (see Supporting Information Figure S10) to confirm they were clean. Afterward, the molecule was dissolved in dichloromethane (DCM) to obtain a solution with a molecular concentration of 0.5 mmol for the *para***-NNR** and 0.2 mmol for the *meta***-NNR**. Approximately 5 μL of this solution was dropcast on the break junction, after which the sample space was closed and pumped to a vacuum below 10^–4^ mbar. Fast-breaking measurements were performed in a vacuum at room temperature, where thousands of conductance vs electrode displacement (breaking) traces were recorded at a constant bias voltage of 100 mV. To measure at low temperatures, the inset with the sample was submerged in liquid helium, cooling the setup down to a temperature of ∼6 K. In this case, current–voltage (*IV*) characteristics were recorded at different electrode displacements in a two-probe configuration: a varying bias voltage is applied across the junction, and the resulting current is measured. Afterward, a Savitzky–Golay filter was applied to the IVs in order to obtain differential conductance vs bias voltage traces.

## Results and Discussion

**Figure 3:**
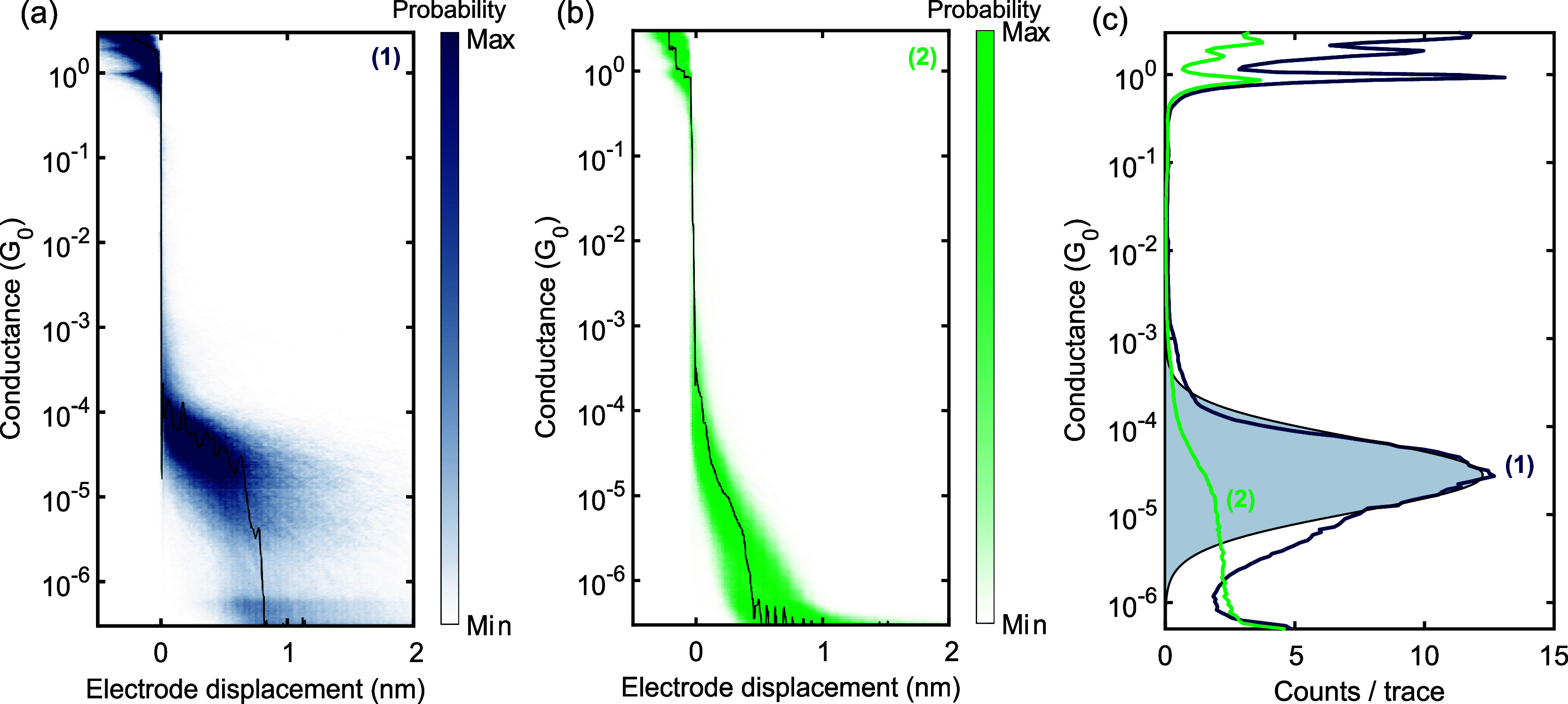
(a) Two-dimensional histogram of the fast-breaking measurements recorded at room temperature in a vacuum on *para***-NNR** constructed from 7.490 consecutive traces measured at a bias voltage of 100 mV. The black line is a single trace from this set. (b) Two-dimensional histogram of the fast-breaking measurements on *meta***-NNR** constructed from 10.000 consecutive traces measured at a bias voltage of 100 mV. (c) Corresponding one-dimensional histogram of the data in (a) and (b). A log-normal distribution (the shaded area) is fitted to the histogram of *para***-NNR** to extract the molecular conductance. For *para***-NNR**, a conductance of 2.8 × 10^–5^*G*_0_ is found.

At room temperature, fast-breaking measurements were performed in a vacuum to obtain statistics on junction formation and molecular conductance. A two-dimensional histogram showing conductance versus electrode displacement is constructed from a set of consecutive breaking traces. Figure 3 displays the two-dimensional histograms for *para***-NNR** (Figure 3a) and *meta***-NNR** (Figure 3b), together with the one-dimensional histogram of both (Figure 3c). A log-normal distribution (shaded area) is fitted to the one-dimensional histogram of *para***-NNR** to obtain the most probable molecular conductance.

The *para***-NNR** displays a clear plateau in the conductance as a function of electrode displacement. The conductance is found to be 2.8 × 10^–5^*G*_0_, where *G*_0_ = 2e^2^/h is the conductance quantum, e is the elementary charge, and h is Planck’s constant. The length of the plateaus is on average ∼0.7 nm. Considering snapback of the gold contacts, this length corresponds with that of the molecule.^18^ The observation of these clear plateaus indicates the formation of molecular junctions. The *meta***-NNR**, on the other hand, does not display clear plateaus at 100 mV; only at higher bias voltages, plateau-like features start to appear (see Supporting Information Figure S11). This indicates that molecular junctions are formed; however, at 100 mV, the plateaus are not visible in the accessible measurement range. The lower conductance of the *meta***-NNR** is consistent with the presence of quantum interference in the central *meta-*connected benzene ring; due to quantum interference, the *meta* connection of the central benzene ring blocks electron waves from passing through it, leading to an overall lower conductance compared to the *para*-connected benzene ring.^14,15^

**Figure 4:**
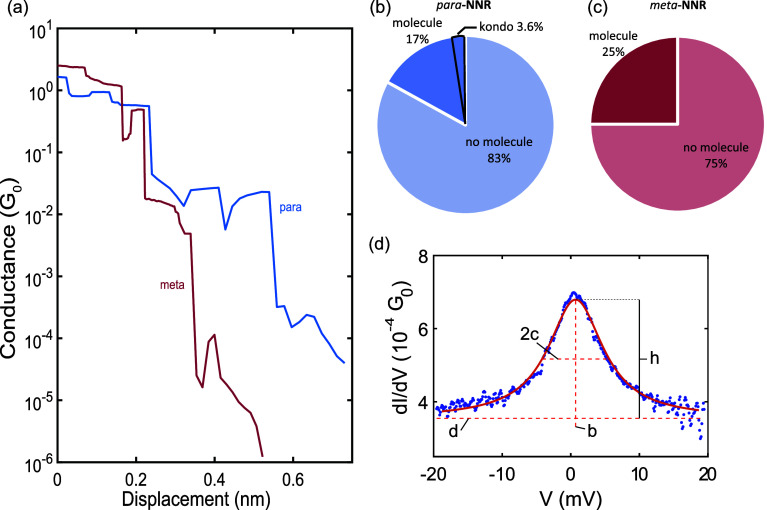
(a) Example of two breaking traces at low temperatures where a molecular junction has formed. The conductance was recorded at a bias voltage of 50 mV. (b,c) Statistics of the breaking measurements at 6 K, showing the percentage of traces which contained a molecular plateau and in the case of the *para***-NNR** how many of those displayed a Kondo peak in the differential conductance. (d) Example of a Kondo peak measured at 6 K with a Lorentzian fitted to it. The blue dots are the recorded data points, and the orange line is the Lorentzian fit. The height *h*, as well as *b*, *c*, and *d* are the fit parameters.

We also studied the electronic transport of *para***-** and *meta***-NNR** at a temperature of around 6 K and recorded current–voltage (*IV*) characteristics while separating the electrodes from each other. For both molecules, plateaus (see Figure 4a for an example) were observed in the conductance between 10^–1^ and 10^–6^*G*_0_, which are attributed to the formation of a molecular junction. There is a large conductance range over which plateaus are observed, possibly due to the high junction stability at cryogenic temperatures, which also leads to the observation of plateaus for *meta***-NNR**. Furthermore, as shown in Figure 4a, the conductance of the plateaus can fluctuate more than 1 order of magnitude within a breaking trace. This is in line with other low-temperature MCBJ experiments and is attributed to the existence of more stable molecular configurations, including those where the electrode distance is smaller than the molecule.^11,13,19^ In the following analysis, we call a plateau molecular if the breaking trace contains more than three data points below a conductance of 1 *G*_0_ and above the noise level. Figure 4b,c shows the statistics for the *para***-** and *meta***-NNR**. For *para***-NNR**, in 17% of the traces, a molecular plateau was observed. For the *meta***-NNR**, this was in 25% of the traces. Inside these plateaus, IVs were recorded to study charge transport through the molecule.

In *para***-NNR**, a small percentage (3.6%) of IVs showed a zero-bias peak in the differential conductance; an example is displayed in Figure 4d. Such a peak has been observed before in molecular junctions where the molecule is a radical^9−11,20−22^ and is attributed to the Kondo effect. Interesting to point out is the study by Zhang et al., where an identical molecule except for the anchoring groups is studied. They observed a Kondo resonance in the antiferromagnetic weak-coupling regime.^21^ While the observed peak is too wide to be split with a magnetic field of 8 T, we did observe a slight suppression of the peak (see Supporting Information Figure S16), consistent with the expectations for a spin- Kondo. To study the width and height of the peak, we fitted a Lorentzian to the zero-bias peaks (Figure 4d):
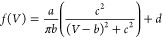
1where *a* is the scaling factor of the height of the peak, *b* is the bias voltage at the maximum of the peak, 2*c* is the full width at half-maximum (FWHM), and *d* is the baseline of the Lorentzian. To address a small degree of asymmetry in some resonances, they were also fitted using a Fano line shape. Both line shapes yielded good fits, but overall the Lorentzian line shape resulted in a higher *R*^2^ and is therefore used in the following discussion (see Supporting Information Section S5 for a comparison between the two line shapes). The width of the peaks was found to be consistently close to 10 meV, independent of the conductance of the molecular junction. This observation is consistent with other studies, where the constant resonance width is explained by an asymmetric coupling between the molecule and the electrodes.^10,11,13^ No trend was observed in the height of the peaks (see Supporting Information Figure S17).

**Figure 5:**
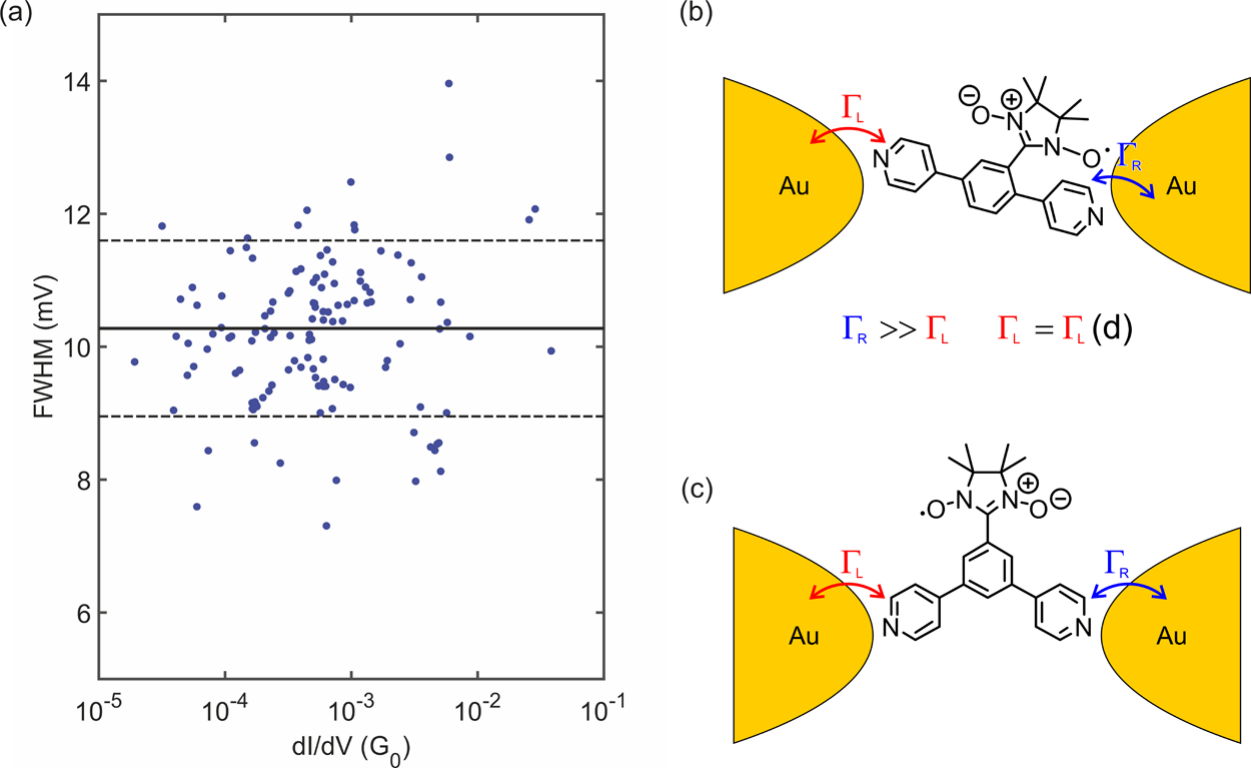
(a) Full width at half-maximum from the Lorentzians fitted to all Kondo peaks versus the conductance of the baseline of the fit (fit parameter *d*). Each blue dot represents an IV measurement. The solid horizontal black line is the average of all values; the dashed black lines are at one standard deviation from the average. Two data points have been left out to improve clarity: one at 16 mV and one at 17 mV but are included in the analysis. (b) Proposed configuration of *para***-NNR** inside the junction. Γ_L_ is the weak coupling of the molecule to the left electrode that changes upon separating the electrodes, and Γ_R_ is the coupling to the right electrode. (c) Proposed configuration of *meta***-NNR** inside the junction.

To observe a Kondo peak, the system temperature needs to be below the characteristic Kondo temperature, *T*_K_, which is related to both the width of the zero-bias peak and the widths of the tunneling barriers between the gold electrodes and the molecule. The FWHM of the peak is related to this Kondo temperature: , where *T* is the sample temperature and *k*_B_ is the Boltzmann constant.^23^ With an average FWHM of 10.3 meV (see Figure 5a) and a temperature of 6 K, we obtain a Kondo temperature of 40.2 K.

Within the Anderson model, the Haldane relation^24^ shows that , where Γ = Γ_*L*_ + Γ_*R*_, the sum of the electronic coupling to the left and right tunneling barriers (Γ_*L*,*R*_), *U* is the Coulomb repulsion energy, and ϵ_0_ is the energy level of the orbital through which electron transport occurs. Under the assumption that only a single level contributes to transport and that the coupling Γ is independent of energy (wide-band limit), the conductance of the molecular junction is . A peak width (i.e., a Kondo temperature) that is constant across several orders of magnitude in the conductance thus suggests that the Γ on one side is much larger than the one on the other side and that only the smaller Γ is sensitive to the separation of both electrodes.^11,13,25^ The former determines the Kondo temperature, whereas the latter determines the conductance of the molecule.

To some extent, this strongly asymmetric coupling to both electrodes was expected for *para***-NNR**. The nitronyl nitroxide radical group interacts much more strongly with the pyridyl anchor group in *ortho*-position compared to the other one to which it is in a *meta* relationship. However, the large difference in the mechanical stabilities of both anchor groups points to an additional stabilization of the *ortho*-pyridyl anchor group. The spatial proximity of the nitronyl nitroxide radical to the *ortho*-pyridyl suggests that the nitronyl nitroxide coordinates directly to the electrode. The hypothesized arrangement is sketched in Figure 5b and would explain the finding of a radical group coupling strongly to only one of the two electrodes, which is at the same time considerably less affected mechanically by variations in the electrode spacing. We thus argue that the conductance through *para***-NNR** is dominated on one side by the radical group, whose connection to the electrode is additionally stabilized by the proximity of the anchoring group (Figure 5b).

Following these arguments, the complete absence of a Kondo peak in *meta-***NNR** would indicate that the radical group is not in direct contact with any of the two electrodes (see the sketch in Figure 5c). Apparently, the electronic contact between the electrodes and the radical group is too weak to lead to observable Kondo features at accessible temperatures. While this can easily be rationalized by the spatial distance between the anchor group and the radical group, it is noteworthy that the effect is additionally assisted by the poor communication through the *meta*-connections between radical and anchor groups in *meta-***NNR**. Both effects lead to a much lower *T*_*K*_, to a value that is below the 6 K reachable in the experiment.

## Conclusions

In conclusion, we have investigated charge transport in a *para*- and *meta*-configured **NNR** in an MCBJ setup through fast-breaking measurements at room temperature and through IVs at 6 K. At room temperature, a clear conductance plateau was observed for the *para***-NNR**, while this was not the case for the *meta***-NNR**. A Kondo peak was observed in the low-temperature measurements on the *para***-NNR**. Analysis of this peak revealed a constant peak width, independent of the conductance of the molecular junction. We hypothesize that this is a result of a very asymmetric coupling of the molecule to the electrodes. The coupling of the radical group to one of the electrodes is stabilized by the proximity of the anchoring group. It would be of interest to perform ab initio conductance calculations considering the different configurations of the molecules inside the junction and to elucidate the role of the stabilization by the anchoring group. These results provide a better understanding of the effect the molecular structure has on electron pathways and the presence of magnetic fingerprints in it. Both are important considerations for creating molecular spintronic devices.

## Supporting Information Available

Synthesis and characterization of *meta***-NNR**;reference measurements on bare gold junctions; fast-breaking measurements on *meta*-**NNR** at higher bias voltages; clustering of the fast-breaking measurements on *meta*-**NNR**; additional fits of the Kondo peaks with a Lorentzian and Fano line shape; magnetic field measurements on *para*-**NNR**; and analysis of the height of the Kondo peaks (PDF)

## References

[ref1] Sanvito S. Molecular spintronics. Chem. Soc. Rev. 2011, 40, 3336 10.1039/c1cs15047b. 21552606

[ref2] Rocha A. R.; García-suárez V. M.; Bailey S. W.; Lambert C. J.; Ferrer J.; Sanvito S. Towards molecular spintronics. Nat. Mater. 2005, 4, 335–339. 10.1038/nmat1349. 15750597

[ref3] Gobbi M.; Novak M. A.; Del Barco E. Molecular spintronics. J. Appl. Phys. 2019, 125, 240401 10.1063/1.5113900.

[ref4] Sangtarash S.; Sadeghi H. Radical enhancement of molecular thermoelectric efficiency. Nanoscale Advances 2020, 2, 1031–1035. 10.1039/C9NA00649D. 36133063 PMC9418312

[ref5] Hurtado-Gallego J.; Sangtarash S.; Davidson R.; Rincón-García L.; Daaoub A.; Rubio-Bollinger G.; Lambert C. J.; Oganesyan V. S.; Bryce M. R.; Agraït N.; Sadeghi H. Thermoelectric Enhancement in Single Organic Radical Molecules. Nano Lett. 2022, 22, 948–953. 10.1021/acs.nanolett.1c03698. 35073099

[ref6] Naghibi S.; Sangtarash S.; Kumar V. J.; Wu J.; Judd M. M.; Qiao X.; Gorenskaia E.; Higgins S. J.; Cox N.; Nichols R. J.; Sadeghi H.; Low P. J.; Vezzoli A. Redox-Addressable Single-Molecule Junctions Incorporating a Persistent Organic Radical**. Angew. Chem. 2022, 134, 202116985 10.1002/ange.202116985. PMC9322687 35289977

[ref7] Sil A.; Hamilton L.; Morris J. M. F.; Daaoub A. H. S.; Burrows J. H. H.; Robertson C. M.; Luzyanin K.; Higgins S. J.; Sadeghi H.; Nichols R. J.; Sangtarash S.; Vezzoli A. Zero-Bias Anti-Ohmic Behaviour in Diradicaloid Molecular Wires. Angew. Chem., Int. Ed. 2024, 63, 202410304 10.1002/anie.202410304. 39003723

[ref8] Li L.; Low J. Z.; Wilhelm J.; Liao G.; Gunasekaran S.; Prindle C. R.; Starr R. L.; Golze D.; Nuckolls C.; Steigerwald M. L.; Evers F.; Campos L. M.; Yin X.; Venkataraman L. Highly conducting single-molecule topological insulators based on mono- and di-radical cations. Nat. Chem. 2022, 14, 1061–1067. 10.1038/s41557-022-00978-1. 35798950

[ref9] Yu L. H.; Natelson D. The Kondo Effect in C 60 Single-Molecule Transistors. Nano Lett. 2004, 4, 79–83. 10.1021/nl034893f.

[ref10] Parks J. J.; Champagne A. R.; Hutchison G. R.; Flores-Torres S.; Abruña H. D.; Ralph D. C. Tuning the Kondo Effect with a Mechanically Controllable Break Junction. Phys. Rev. Lett. 2007, 99, 026601 10.1103/PhysRevLett.99.026601. 17678242

[ref11] Frisenda R.; Gaudenzi R.; Franco C.; Mas-Torrent M.; Rovira C.; Veciana J.; Alcon I.; Bromley S. T.; Burzurí E.; van der Zant H. S. J. Kondo Effect in a Neutral and Stable All Organic Radical Single Molecule Break Junction. Nano Lett. 2015, 15, 3109–3114. 10.1021/acs.nanolett.5b00155. 25897770

[ref12] Hayakawa R.; Karimi M. A.; Wolf J.; Huhn T.; Zöllner M. S.; Herrmann C.; Scheer E. Large Magnetoresistance in Single-Radical Molecular Junctions. Nano Lett. 2016, 16, 4960–4967. 10.1021/acs.nanolett.6b01595. 27458666

[ref13] Mitra G.; Low J. Z.; Wei S.; Francisco K. R.; Deffner M.; Herrmann C.; Campos L. M.; Scheer E. Interplay between Magnetoresistance and Kondo Resonance in Radical Single-Molecule Junctions. Nano Lett. 2022, 22, 5773–5779. 10.1021/acs.nanolett.2c01199. 35849010

[ref14] Arroyo C. R.; Frisenda R.; Moth-Poulsen K.; Seldenthuis J. S.; Bjo̷rnholm T.; van der Zant H. S. Quantum interference effects at room temperature in OPV-based single-molecule junctions. Nanoscale Res. Lett. 2013, 8, 234 10.1186/1556-276X-8-234. 23679986 PMC3663707

[ref15] Yang G.; Wu H.; Wei J.; Zheng J.; Chen Z.; Liu J.; Shi J.; Yang Y.; Hong W. Quantum interference effect in the charge transport through single-molecule benzene dithiol junction at room temperature: An experimental investigation. Chin. Chem. Lett. 2018, 29, 147–150. 10.1016/j.cclet.2017.06.015.

[ref16] Pyurbeeva E.; Hsu C.; Vogel D.; Wegeberg C.; Mayor M.; van der Zant H.; Mol J. A.; Gehring P. Controlling the Entropy of a Single-Molecule Junction. Nano Lett. 2021, 21, 9715–9719. 10.1021/acs.nanolett.1c03591. 34766782

[ref17] Martin C. A.; Smit R. H. M.; van Egmond R.; van der Zant H. S. J.; van Ruitenbeek J. M. A versatile low-temperature setup for the electrical characterization of single-molecule junctions. Rev. Sci. Instrum. 2011, 82, 053907 10.1063/1.3593100. 21639518

[ref18] Hong W.; Manrique D. Z.; Moreno-García P.; Gulcur M.; Mishchenko A.; Lambert C. J.; Bryce M. R.; Wandlowski T. Single Molecular Conductance of Tolanes: Experimental and Theoretical Study on the Junction Evolution Dependent on the Anchoring Group. J. Am. Chem. Soc. 2012, 134, 2292–2304. 10.1021/ja209844r. 22175273

[ref19] Frisenda R.; Stefani D.; van der Zant H. S. J. Quantum Transport through a Single Conjugated Rigid Molecule, a Mechanical Break Junction Study. Acc. Chem. Res. 2018, 51, 1359–1367. 10.1021/acs.accounts.7b00493. 29862817

[ref20] Temirov R.; Lassise A.; Anders F. B.; Tautz F. S. Kondo effect by controlled cleavage of a single-molecule contact. Nanotechnology 2008, 19, 065401 10.1088/0957-4484/19/6/065401. 21730697

[ref21] Zhang Y.-h.; Kahle S.; Herden T.; Stroh C.; Mayor M.; Schlickum U.; Ternes M.; Wahl P.; Kern K. Temperature and magnetic field dependence of a Kondo system in the weak coupling regime. Nat. Commun. 2013, 4, 2110 10.1038/ncomms3110. 23817525 PMC3730050

[ref22] Baum T. Y.; Fernández S.; Peña D.; van der Zant H. S. J. Magnetic Fingerprints in an All-Organic Radical Molecular Break Junction. Nano Lett. 2022, 22, 8086–8092. 10.1021/acs.nanolett.2c02326. 36206381 PMC9614975

[ref23] Nagaoka K.; Jamneala T.; Grobis M.; Crommie M. F. Temperature Dependence of a Single Kondo Impurity. Phys. Rev. Lett. 2002, 88, 077205 10.1103/PhysRevLett.88.077205. 11863936

[ref24] Haldane F. D. M. Scaling Theory of the Asymmetric Anderson Model. Phys. Rev. Lett. 1978, 40, 416–419. 10.1103/PhysRevLett.40.416.

[ref25] Appelt W. H.; Droghetti A.; Chioncel L.; Radonjić M. M.; Muñoz E.; Kirchner S.; Vollhardt D.; Rungger I. Predicting the conductance of strongly correlated molecules: the Kondo effect in perchlorotriphenylmethyl/Au junctions. Nanoscale 2018, 10, 17738–17750. 10.1039/C8NR03991G. 30211420

