# Supplementary material for: Mechanically Stable Kondo Resonance in an Organic Radical Molecular Junction
Source: J Phys Chem C Nanomater Interfaces. 2025 Jan 30;129(6):3152–7. doi: 10.1021/acs.jpcc.4c05860 (PMC11831679; doi:10.1021/acs.jpcc.4c05860)
Supplement: Supplementary file 1 — jp4c05860_si_001.pdf [file jp4c05860_si_001.pdf]

Mechanically Stable Kondo Resonance in an Organic Radical Molecular Junction Supporting Information

Tristan Bras,† Chunwei Hsu,† Thomas Baum,† David Vogel,‡ Marcel Mayor,‡ and Herre S.J. van der Zant*,†

†*Kavli Institute of Nanoscience, Delft University of Technology, Lorentzweg 1, Delft 2628 CJ, The Netherlands*

‡*Department of Chemistry, University of Basel, St. Johanns-Ring 19, 4056 Basel, Switzerland*

E-mail: h.s.j.vanderzant@tudelft.nl

Contents

S1 Synthesis **S2**

S2 Reference measurements on bare gold junctions **S11**

S3 Two-dimensional histograms of *meta*-NNR at different bias voltages **S12**

S4 Clustering of the *meta*-NNR fast-breaking measurements **S12**

S5 Fits on Kondo peaks **S14**

S6 Magnetic field measurements on *para*-NNR **S17**

S7 Height of the Kondo resonances **S18**

References **S19**

S1 Synthesis

General Remarks: All chemicals and anhydrous solvents were used as purchased without further purification, unless stated otherwise. Deuterated solvents were obtained from Cambridge Isotope Laboratories, Inc. (Andover, MA, USA). All other commercially available starting materials and solvents were purchased from Honeywell, Sigma-Aldrich, Acros or Fluorochem. NMR experiments were acquired on a 400 or 500 MHz Bruker Avance III spectrometer equipped with a QNP or BBFO probe head respectively. All spectra were recorded at 298 K. The chemical shifts (δ) are reported in parts per million (ppm) relative to tetramethylsilane or referenced to residual solvent peaks and the J values are given in Hz ($\pm$ 0.1 Hz). EPR spectra (X-band) were recorded on a Bruker ELEXSYS-II E500 CW-EPR spectrometer. Samples for EPR measurements were prepared in capillary tubes and measured at r.t. For high-resolution mass spectrometry (HRMS) a HR-ESI-ToF-MS measurement on a maXisTM 4G instrument from Bruker was performed. Column chromatography was performed on SiliaFlash®P60 from SILICYCLE with a particle size of 40-63 µm (230-400 mesh). Thin layer chromatography (TLC) was performed on Silica gel 60 F254 glass plates with a thickness of 0.25 mm from Merck using fluorescent quenching under UV light at 254 nm for the localization of sample spots.

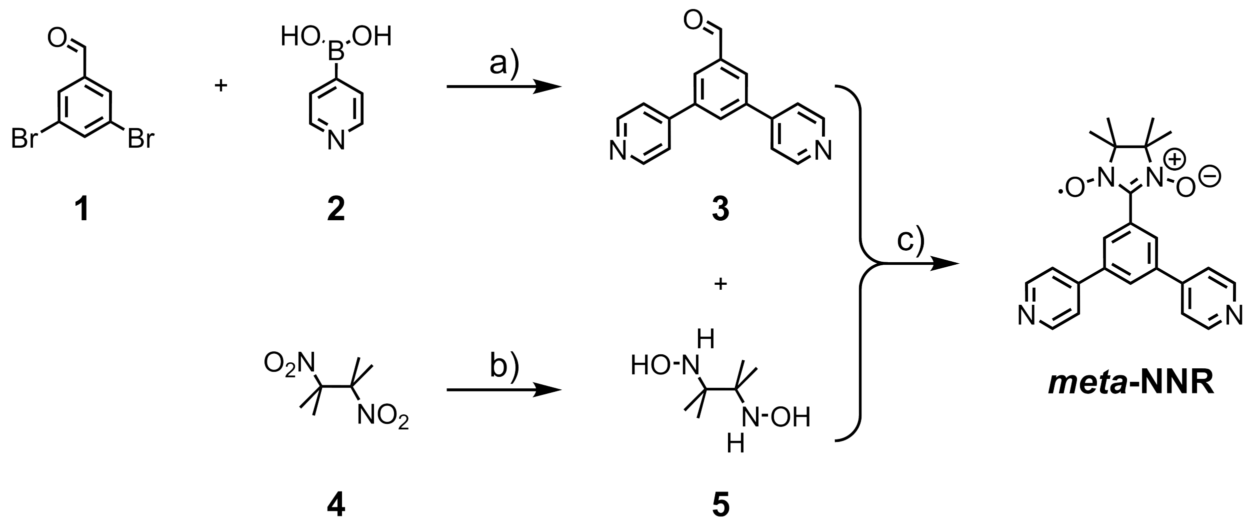

Figure S1: a) Pd(PPh_3)$_2$Cl$_2$, K_2CO_3, dioxane, MeOH, 90 °C, 6 h, 74%; b) Zn, NH_4Cl, THF, H_2O, 0 - 10 °C, 48 h, 57%; c) 1) MeOH, r.t., 2d, 2) PbO_2, overnight, r.t., 31%.

Double Suzuki cross-coupling of 3,5-bibromobenzaldehyd **1** with 4-pyridine boronic acid **2** yielded the literature known 3,5-di(pyridin-4-yl) benzaldehyde **3** in good yield of 74%.[1] Next, the hydroxylamine **5** was prepared by a reduction of the corresponding nitro derivative **4** with zinc following a literature known protocol.[2] Thereafter a condensation of 3,5-di(pyridin-4-yl) benzaldehyde **3** and hydroxylamine **5** yielded the desired radical precursor, which due to stability reasons was directly oxidized with lead dioxide to the desired nitronyl nitroxide radical ***meta*-NNR** in a moderate yield of 31%.

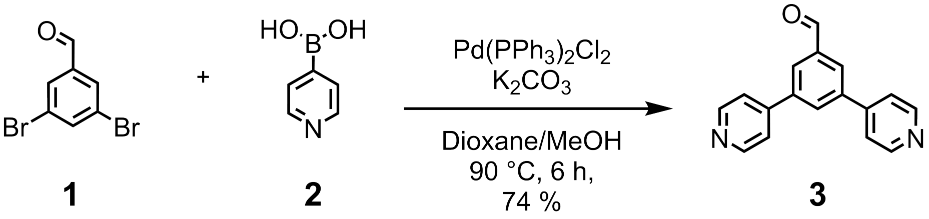

Figure S2: Synthesis of 3,5-di(pyridin-4-yl)benzaldehyde **3**.

3,5-di(pyridin-4-yl)benzaldehyde 3: An oven dried Schlenk flask (50 mL) was charged with 3,5-bibromobenzaldehyd **1** (1.0 g, 3.79 mmol, 1 eq.), 4-pyridinboronic acid **2** (1.14 g, 8.34 mmol, 2.2 eq.) and K_2CO_3(2.31 g, 16.7 mmol, 4.4 eq.) and degassed. Then MeOH (10 mL) and Dioxan (20 mL) were added to the flask and degassed for 15 minutes. Then

$Pd(PPh_3)_2Cl_2$ (94.8 mg, 0,135 mmol, 0,06 eq.) was added and the resulting mixture was stirred under Argon at 90 °C for 6 h. The reaction mixture was then diluted with an aqueous saturated sodium carbonate solution and extracted twice with DCM. The organic phase was then dried over Mg_2SO_4, filtered and concentrated under reduced pressure. The crude product was then further purified by column chromatography on silica gel (cyclohexan/EtOAc: 4/1) yielding aldehyde **3** as an off white solid (741 mg, 3.79 mmol, 74%). The obtained analytical data matched previously reported data.[1]

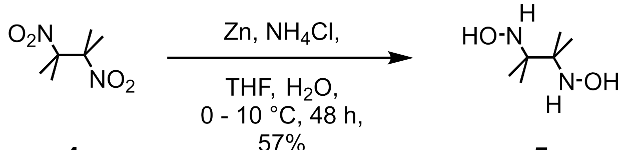

4 **5**

Figure S3: Synthesis of N,N'-(2,3-dimethylbutane-2,3-diyl)bis(hydroxylamine) **5**.

N,N'-(2,3-dimethylbutane-2,3-diyl)bis(hydroxylamine) 5: A round bottom flask was charged with 2,3-dimethyl-2,3-dinitrobutane **4** (5 g, 27.8 mmol,1 eq.), tetrahydrofuran (86 mL) and water (14 mL). This solution was then cooled in an ice bath and degassed with argon (15 min). Thereafter zinc powder (7.6 g, 117 mmol, 4.2 eq.) was added at once. Then a solution of NH_4Cl (12.5 g, 234 mmol, 8.4 eq.) in water (42 mL) was added dropwise over a duration of 20 min. The reaction mixture was then stirred at 10 °C for 1 h and placed in the fridge over night for 18 h. The suspension was then filtered, and the filter cake was washed four times with THF (4x 25 mL). The collected filtrate was then concentrated in vaccuo resulting in a syrupy like mixture. To this sirup was added Na_2CO_3 (11 g), NaCl (7 g) and Na_2SO_4 (7 g). The resulting mud like mixture was then transferred to a soxlet, protected from air and continuously extracted with DCM (90 mL), yielding compound **5** as an off white solid (2.330 g, 15.7 mmol, 57%). The hydroxylamine **5** degrades fast at r.t., slower at 0 °C and is stable for a few months at -20 °C. The obtained analytical data matched previously reported data.[2]

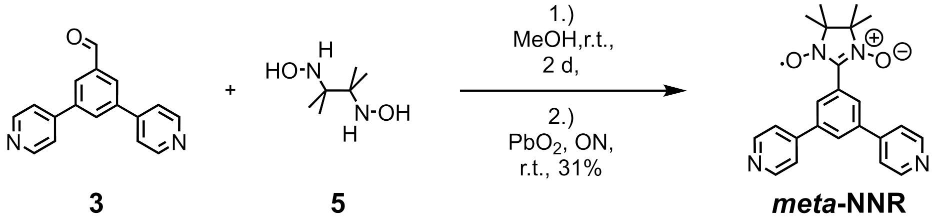

Figure S4: Synthesis of 2-(3,5-di(pyridin-4-yl)phenyl)-1-hydroxy-4,4,5,5-tetramethyl-4,5-dihydro-1H-imidazole 3-oyl radical ***meta*-NNR**.

2-(3,5-di(pyridin-4-yl)phenyl)-1-hydroxy-4,4,5,5-tetramethyl-4,5-dihydro- 1H-imidazole 3-oyl radical (*meta*-NNR): An oven dried argon flushed round bottom flask was charged with the aldehyde **3** (300 mg, 1.15 mmol, 1 eq.), hydroxylamine **5** (256 mg, 1.72 mmol, 1.5 eq.) and degassed. Then dry MeOH (20 mL) was added and the reaction mixture was stirred in the dark for 2 d. Then PbO_2 was added and the reaction mixture was again stirred overnight. Then potassium carbonate (spatula tip) was added and stirred for 1 h. The crude mixture was filtered and concentrated under reduced pressure (rotational evaporator, water bath temperature: 30 °C). The crude product was purified by column chromatography on silica gel (DCM/ACN: 20/1) followed by prep.HPLC on silica gel (DCM/MeOH: 19/1) yielding ***meta*-NNR** as a blue solid (139 mg, 1.15 mmol, 31%).

HRMS (ESI): m/z: calcd. for $[C_23H_23N_4O_2+H]^+$ 388.1901; $[M+H]^+$ found 388.1894.

EPR (X-band): g-value = 2.0065 with a spacing of a_N = 7.10 G

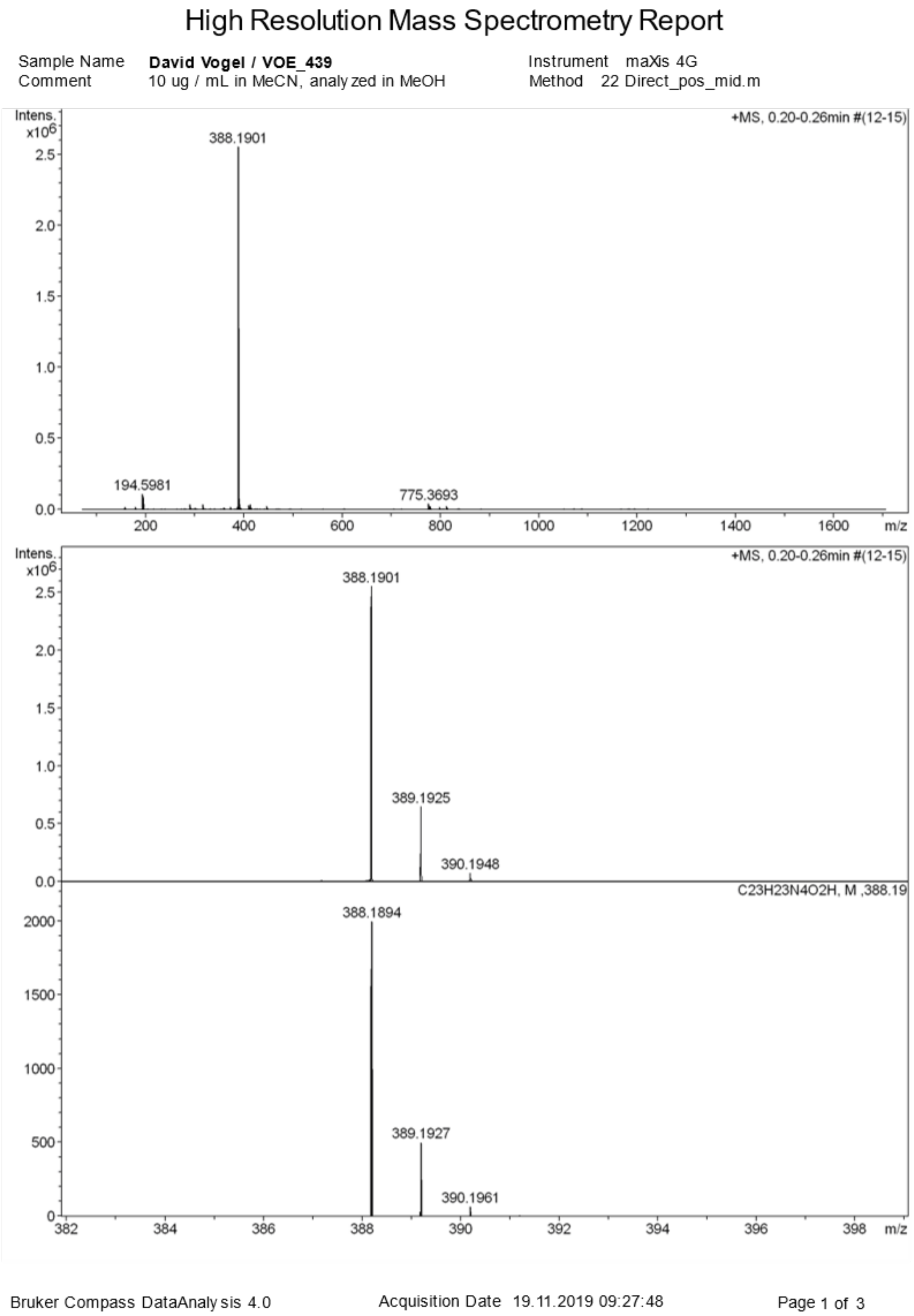

Figure S5: HRMS (ESI) spectrum of ***meta*-NNR**.

High Resolution Mass Spectrometry Report

Measured m/z vs. theoretical m/z

Meas. m/z	#	Formula	Score	m/z	err [mDa]	err [ppm]	mSigma	rdb	e⁻ Conf	z
388.1901	1	C 23 H 24 N 4 O 2	100.00	388.1894	-0.7	-1.8	6.4	14.0	odd	1+

Mass list

#	m/z	I %	I
1	157.0762	0.2	4696
2	158.5757	0.7	17871
3	159.0771	0.1	3826
4	179.5989	0.7	17859
5	180.1009	0.2	5158
6	194.5981	4.3	110166
7	195.0995	1.1	28421
8	195.6011	0.2	4318
9	205.0594	0.2	3963
10	217.1041	0.2	5491
11	233.1063	0.3	7703
12	264.0883	0.2	4317
13	273.1669	0.2	4324
14	277.2131	0.1	3489
15	279.1573	0.1	3512
16	279.2285	0.2	4877
17	291.1120	1.5	38099
18	292.1154	0.3	7051
19	293.2079	0.2	4869
20	297.2391	0.2	4225
21	301.1392	0.5	13811
22	302.1560	0.2	4640
23	303.1771	0.2	4541
24	305.2442	0.1	3741
25	316.1438	1.5	37287
26	317.1468	0.3	8243
27	319.2237	0.2	4591
28	321.2388	0.2	3921
29	331.2083	0.2	4014
30	333.1596	0.2	5281
31	341.2651	0.2	4567
32	353.2655	0.2	5915
33	358.1907	0.4	10771
34	359.1942	0.1	3511
35	361.1648	0.4	10339
36	372.1935	0.8	20733
37	373.1986	0.3	8000
38	381.2968	0.1	3745
39	385.2915	0.2	4110
40	387.1805	0.7	17851
41	388.0760	0.3	6436
42	388.1626	0.9	22973
43	388.1901	100.0	2554552
44	388.4115	0.2	4475
45	388.4306	0.2	3993
46	388.4998	0.2	5180
47	388.5924	0.1	3543
48	388.6830	0.2	5704
49	389.1925	25.4	649699
50	390.1948	3.1	79660
51	391.1975	0.3	8456
52	391.2835	0.4	9109
53	393.2965	0.4	9939
54	410.1704	1.2	30068
55	411.1733	0.3	8872
56	413.2653	1.5	37861
57	414.2688	0.4	10342
58	421.3278	0.2	3896
59	425.2137	0.2	4700
60	425.3610	0.2	5705
61	427.2081	0.2	5427
62	433.1022	0.2	5733

Bruker Compass DataAnalysis 4.0 Acquisition Date 19.11.2019 09:27:48 Page 2 of 3

Figure S6: HRMS (ESI) peak table of ***meta*-NNR** part 1.

High Resolution Mass Spectrometry Report

#	m/z	I %	I
63	441.2964	0.3	6524
64	447.3436	1.1	27155
65	448.3469	0.3	8365
66	449.3593	0.2	4619
67	469.3273	0.2	3834
68	473.3439	0.1	3605
69	492.2143	0.2	5290
70	495.2057	0.2	5211
71	517.3707	0.2	3942
72	603.3058	0.2	6204
73	705.5807	0.2	3939
74	721.5739	0.2	4247
75	775.3693	1.6	40285
76	776.3725	0.9	22586
77	777.3748	0.3	6825
78	778.4642	0.9	21885
79	779.4671	0.4	11292
80	780.4694	0.1	3821
81	797.3512	0.6	16429
82	798.3533	0.4	9005
83	803.5399	0.1	3744
84	806.4944	0.2	5805
85	807.4995	0.1	3635
86	812.5422	0.9	23753
87	813.5451	0.5	14012
88	814.5503	0.2	5840
89	834.5247	0.2	4903
90	837.6199	0.2	5042
91	882.3860	0.2	4516
92	1050.6304	0.2	4015
93	1070.7772	0.2	5397
94	1071.7805	0.2	4250
95	1086.7691	0.2	5800
96	1087.7745	0.2	4009
97	1166.8860	0.2	4162
98	1180.9022	0.1	3793
99	1192.9000	0.2	3933
100	1194.9177	0.2	4602

Acquisition Parameter

**General**	Fore Vacuum	2.69e+000 mBar	High Vacuum	8.71e-008 mBar	Source Type	ESI
	Scan Begin	75 m/z	Scan End	1700 m/z	Ion Polarity	Positive
**Source**	Set Nebulizer	0.4 Bar	Set Capillary	3600 V	Set Dry Gas	4.0 l/min
	Set Dry Heater	180 °C	Set End Plate Offset	-500 V		
**Quadrupole**	Set Ion Energy (MS only)	4.0 eV				
**Coll. Cell**	Collision Energy	8.0 eV	Set Collision Cell RF	350.0 Vpp		
**Ion Cooler**	Set Ion Cooler Transfer Time	75.0 µs	Set Ion Cooler Pre Pulse Storage Time	10.0 µs		

Bruker Compass DataAnalysis 4.0 Acquisition Date 19.11.2019 09:27:48 Page 3 of 3

Figure S7: HRMS (ESI) peak table of ***meta*-NNR** part 2.

EPR Analysis of *meta*-NNR and *para*-NNR

meta-NNR	*para*-NNR
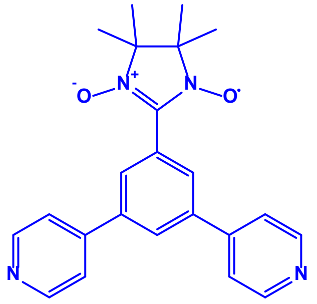	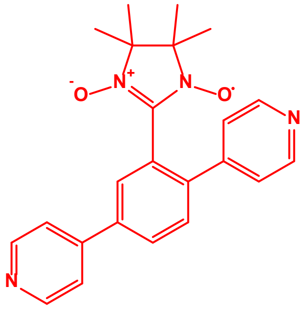

Temperature dependent EPR analysis of *meta*-NNR and *para*-NNR

Conc	*meta*-NNR	*para*-NNR
**0.5 mM**	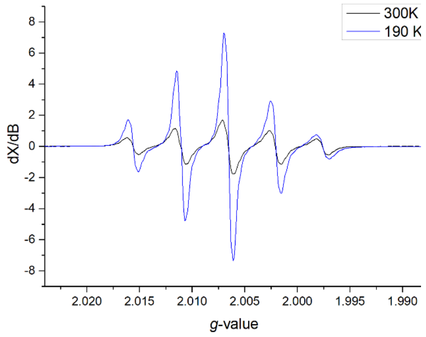	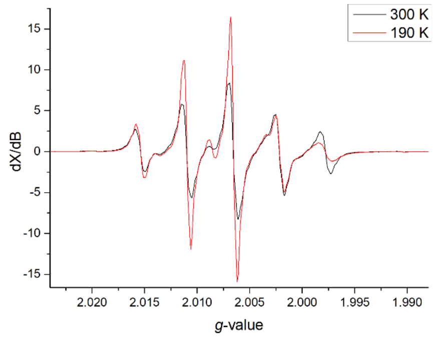
**2.5 mM**	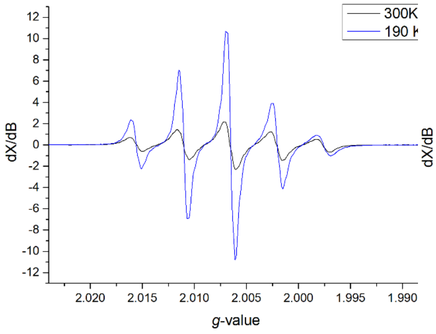	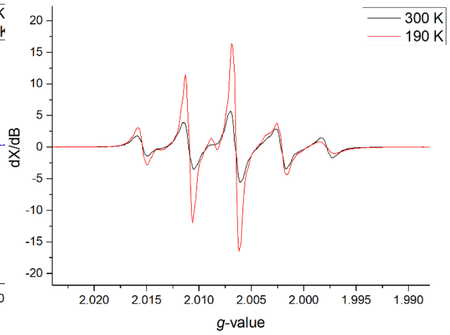

All spectra are recorded on DCM solutions.

Figure S8: Temperature dependent EPR analysis of ***meta*-NNR** and ***para*-NNR**.

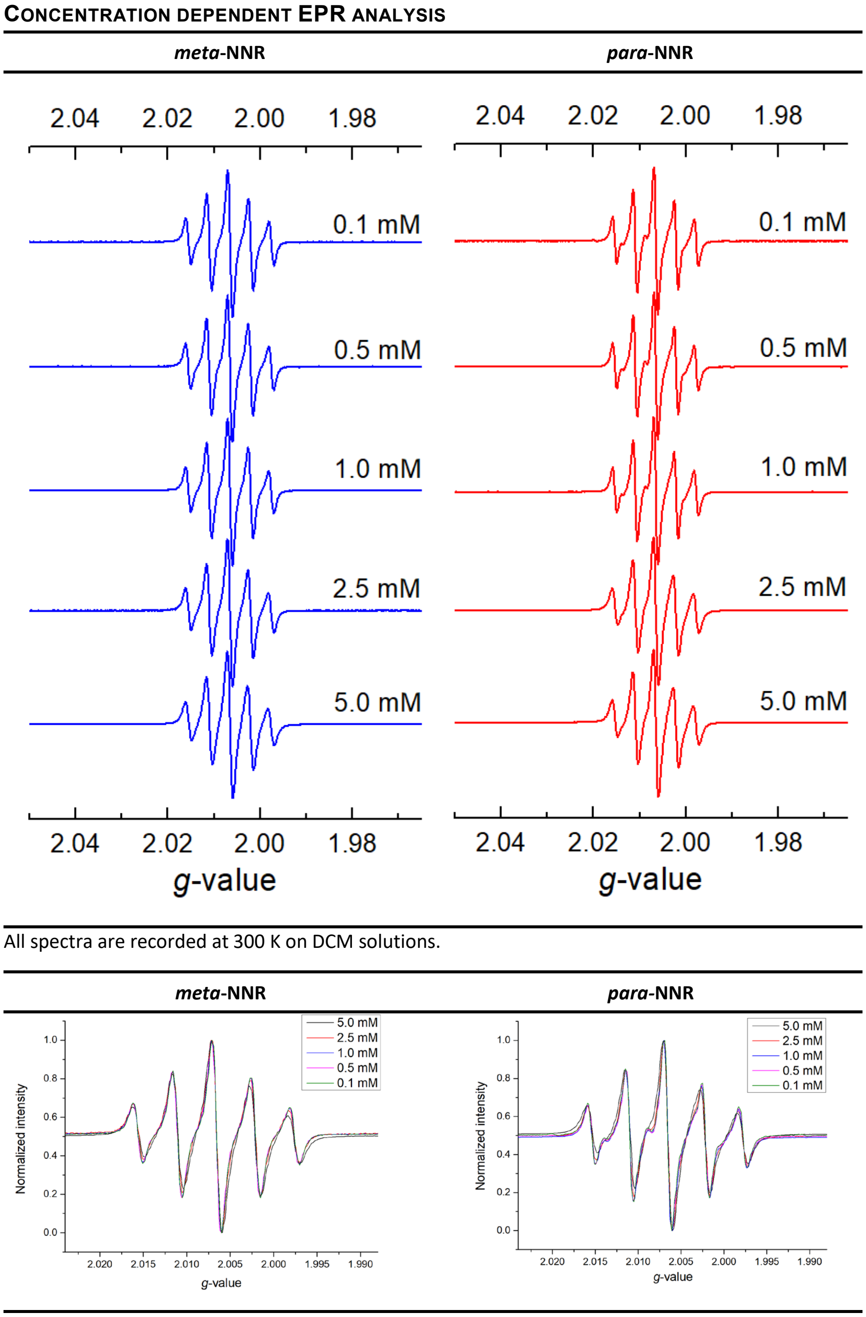

Figure S9: Concentration dependent EPR analysis of ***meta*-NNR** and ***para*-NNR**.

S2 Reference measurements on bare gold junctions

Prior to using a sample for molecular measurements, the sample is characterized. In case no contaminations are present on the sample, an exponential decay of the conductance as a function of electrode separation is expected. Figure S10 shows these measurements performed on the same junctions as those which were used for the measurements displayed in Figure 3 of the main text. In both cases the conductance decays exponentially with increasing electrode displacement, indicating a clean junction.

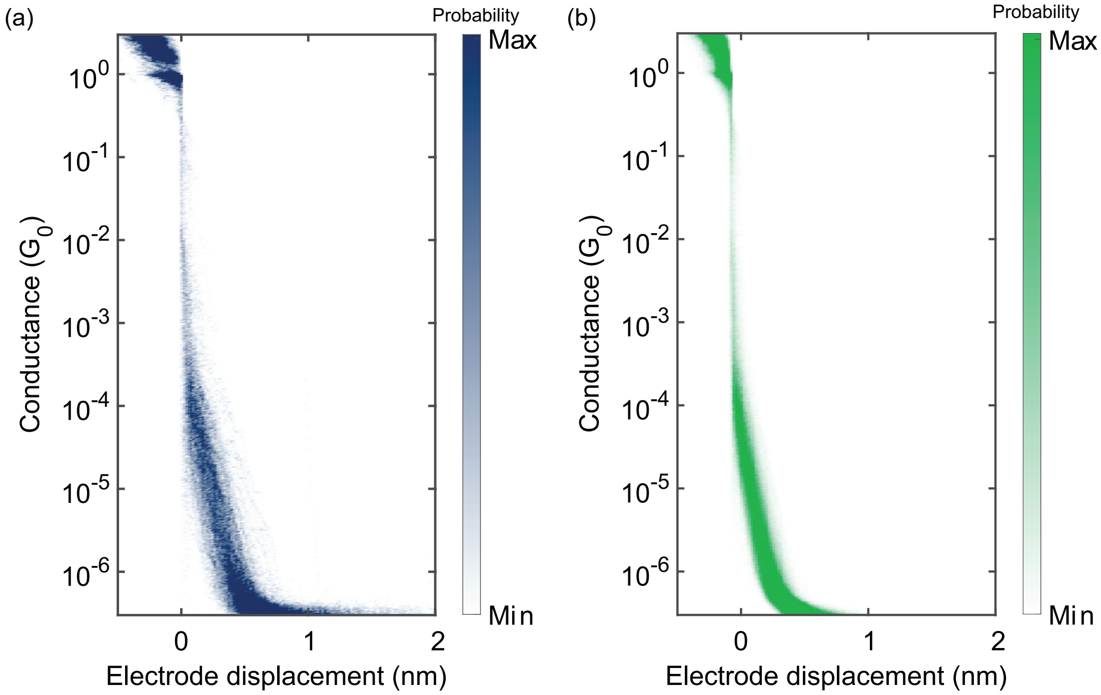

Figure S10: a) Two-dimensional histogram of the fast-breaking measurements on a bare gold junction. The same junction was used for the measurements on the para-nitronyl-nitroxide radical in this paper. Traces were recorded at a bias voltage of 100 mV. b) Two-dimensional histogram of the fast-breaking measurements on a bare gold junction which was later used to measure the meta-nitronyl-nitroxide radical.

S3 Two-dimensional histograms of *meta*-NNR at different bias voltages

In our break junction experiments we record sets of consecutive traces at different bias voltages. For the ***meta*-NNR**, no clear plateaus were observed at a bias voltage of 100 mV (see Fig. 3 in the main text). Figure S11 shows two-dimensional histograms of sets of consecutive breaking traces recorded at 100 mV (a), 250 mV (b) and 350 mV (c). At 100 mV, the histogram is barely distinguishable from the bare gold junction (Fig. S10b). At higher bias, a feature forms at a conductance of around 10^{-5} G_0 as more plateaus are observed.

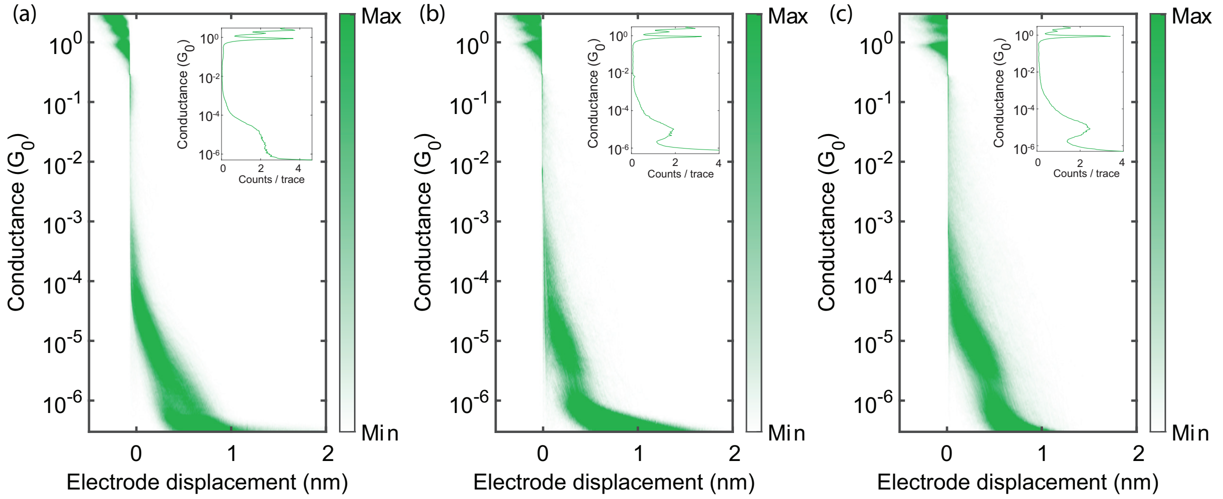

Figure S11: a) Two-dimensional histogram of fast-breaking measurements recorded at room-temprature in a vacuum on the meta-nitronyl-nitroxide radical at a bias voltage of 100 mV. b) At 250 mV. c) At 350 mV.

S4 Clustering of the *meta*-NNR fast-breaking measurements

In figure S11a, around 10^{-6} G_0 two clusters seem to be present: one which decays quickly to the noise level and one which decays slower. To better investigate this, we used a neural network model,[3] which has been trained with datasets of both bare gold junctions and

junctions containing molecules, to separate breaking traces of the fast-breaking measurement recorded at a bias voltage of 100 mV. The result is shown in figure S12. The algorithm was set to consider the region between a conductance of 10^{-2} and 10^{-6} G_0 and between an electrode displacement of 0 and 2.5 nm. The first panel shows the two-dimensional histogram of the traces which are labeled as class 1 and the second panel shows the two-dimensional histogram of the traces which are labeled as class 2. Class 1 has a lower decay factor compared to the one of class 2, most likely due to the presence of molecules on the electrode surface. No conductance plateaus are observed, nor a clear peak in the one-dimensional histogram; hence we cannot assign a most likely molecular conductance to the molecule from this measurement at 100 mV.

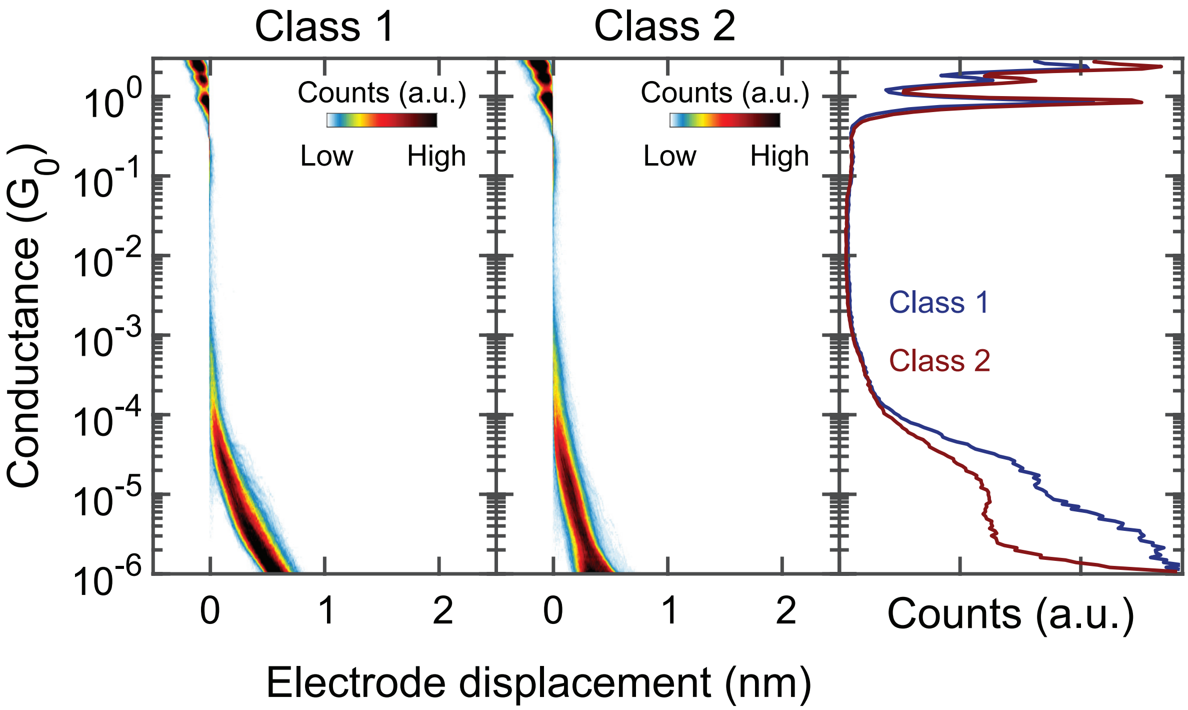

Figure S12: Two-dimensional and one-dimensional histograms obtained after clustering the dataset of the fast-breaking measurements on ***meta*-NNR** recorded at a bias voltage of 100 mV (Fig. S11a).

S5 Fits on Kondo peaks

In total, 129 zero-bias peaks were observed among 3330 IVs. A Lorentzian was fitted to all these peaks in order to determine the width and the height of the peaks. In most cases, the Lorentzian fitted the data accurately. Figure S13 shows several more peaks with the corresponding fit.

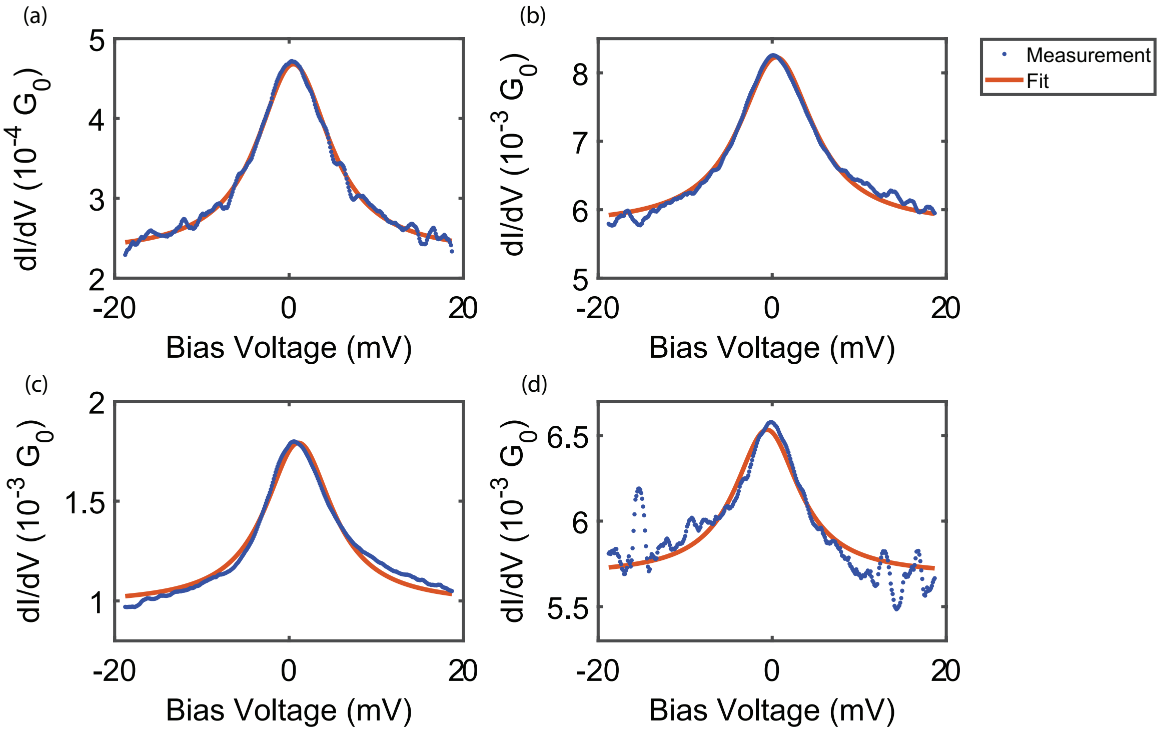

Figure S13: a-d) Four examples of observed zero-bias peaks in the differential conductance as a function of bias voltage (blue). Each of these peaks was fitted with a Lorentzian (orange).

Because several Kondo resonances displayed a small degree of asymmetry, we also fitted the peaks using a Fano lineshape:[4,5]

$$Fano(V) \propto \frac{(q+\epsilon)^2}{1+\epsilon^2}, \tag{1}$$

with q the asymmetry factor and $\epsilon = (eV - E_k)/\Gamma_{Fano}$ where e is the elementary charge $1.6 \cdot 10^{-19}$, V the bias voltage, E_k the energy of the resonance and Γ_{Fano} the half-width at half-maximum of the resonance. Fits of the same resonances as in figure S13 are shown in figure S14. In most cases, good fits could be made, however, as seen in panel d this was not always the case. From the fitting of a Fano lineshape to the 129 Kondo resonances we found an average q factor of 16.6 with a standard deviation of 4.26. For a comparison between the fits with a Lorentzian lineshape and a Fano lineshape, the R^2 values for both are shown in figure S15. It is clear that a Lorentzian lineshape yields better fits.

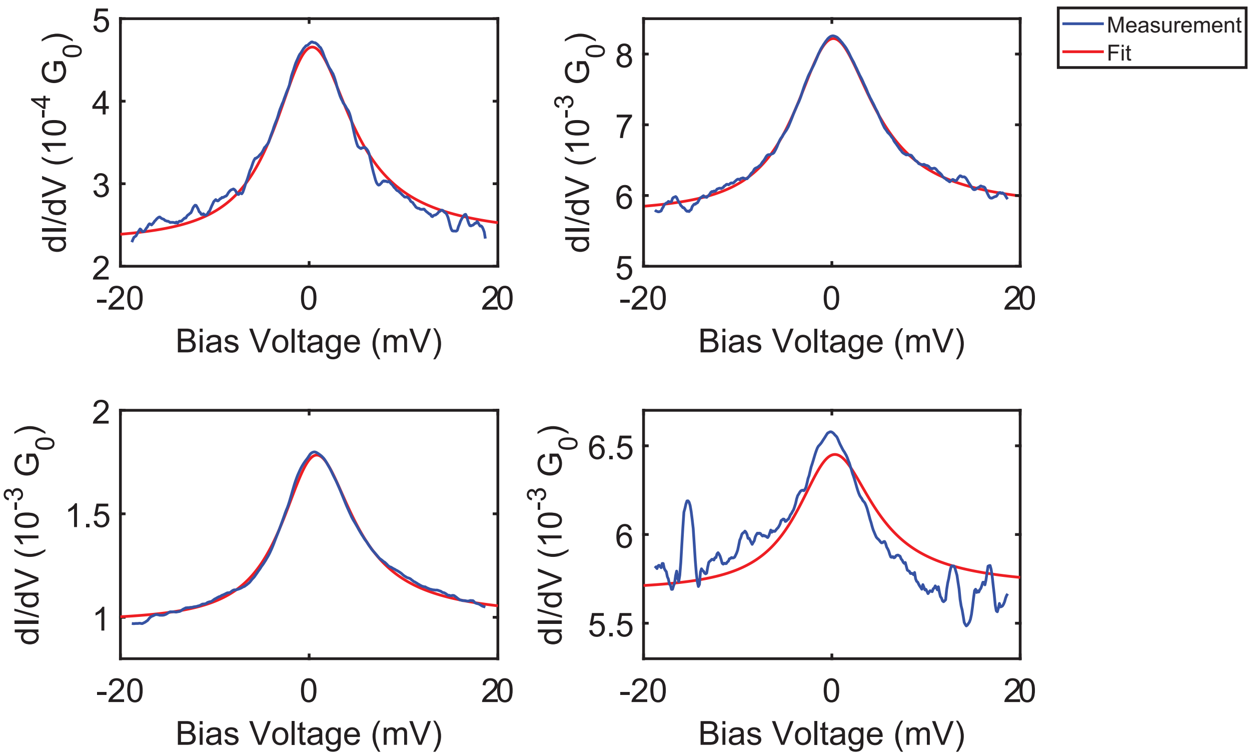

Figure S14: a-d) Four examples of observed zero-bias peaks in the differential conductance as a function of bias voltage (blue). Each of these peaks was fitted with a Fano lineshape (orange).

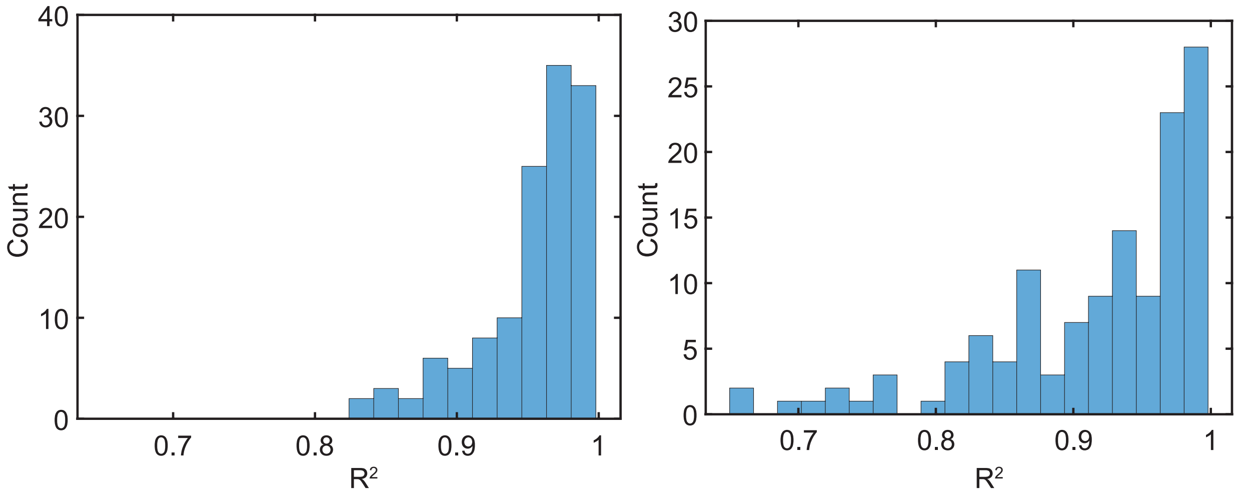

Figure S15: a) Histogram of the squared residuals for the fit using a Lorentzian lineshape. b) Histogram of the squared residuals for the fit using a Fano lineshape.

S6 Magnetic field measurements on *para*-NNR

One key characteristic of a Kondo resonance, is that it will split under a magnetic field due to the lifting of the spin-degeneracy. Depending on the width of the peak (and thus the Kondo temperature), this is observed as either a suppression of the peak, or a full splitting. In the case of the ***para*-NNR**, the peak width is usually around 10 meV, which is too broad to split. In one magnetic field measurement (out of 5) however, a suppression of the peak at high magnetic fields was observed (see Fig. S16). In order to qualitatively view the behavior of the peak, the traces were all normalized with respect to the baseline (parameter d) of the fitted Lorentzian.

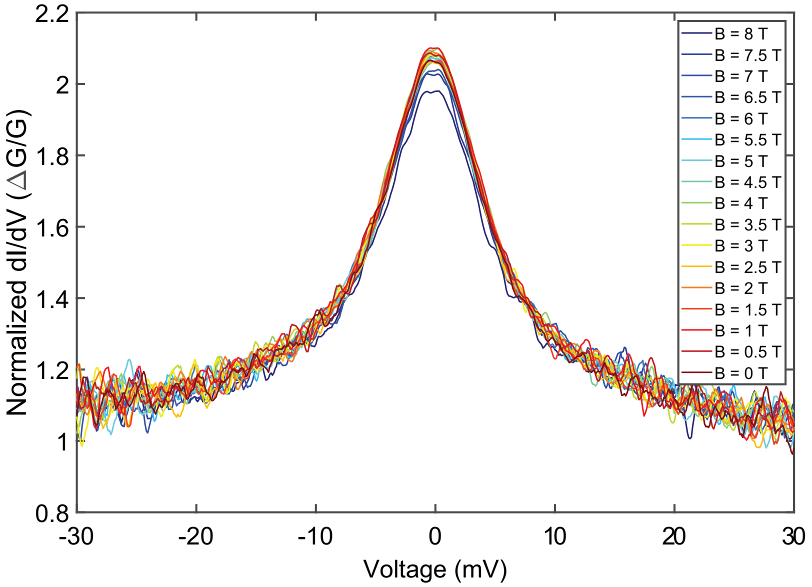

Figure S16: Plot of the normalized differential conductance as a function of bias voltage at magnetic fields ranging from 0 T to 8 T measured on the para-nitronyl-nitroxide radical. The conductance was normalized with respect to the baseline (parameter d) of the fitted Lorentzian.

S7 Height of the Kondo resonances

The height of the peaks was analyzed in the same way as the width. The height of a peak was defined as the difference between the highest point of the peak and the baseline of the fitted Lorentzian. In order to compare traces which were recorded at different order of magnitude of conductance, the height was normalized with respect to the baseline of the fitted Lorentzian. This resulted in figure S17. No trend is observed: there is no correlation between the height of the peak and the conductance at which it was recorded, nor is it constant across different orders of magnitude of conductance. The large variation in normalized height is probably due to the changing coupling strength of the backbone of the molecule during breaking. The lack of correlation suggests that many different configurations are possible, between which the coupling of the backbone to the electrodes and the radical group to the electrodes do not depend on each other.

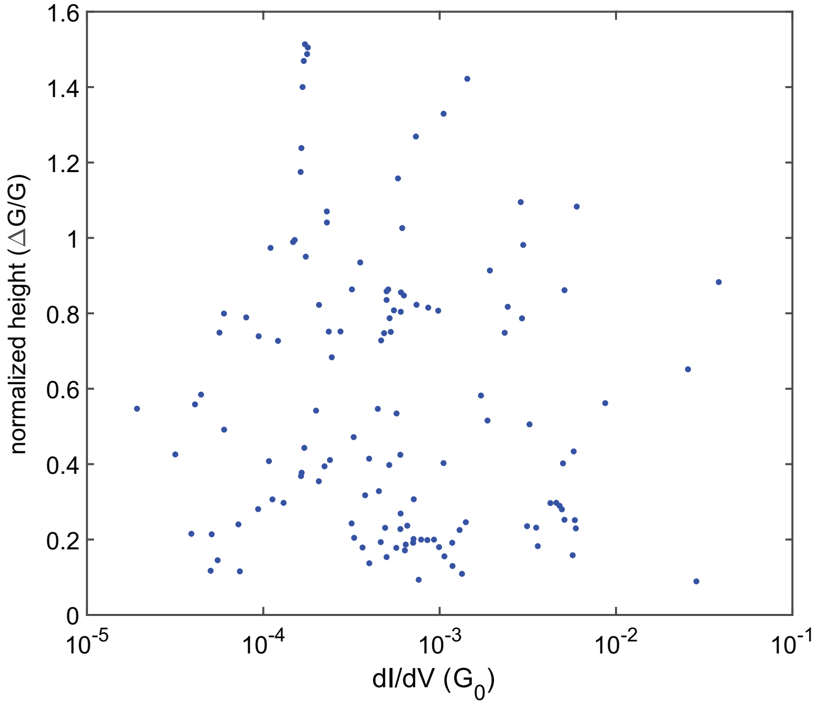

Figure S17: The normalized height of the Lorentzians fitted to differential conductance measurements taken on the para-nitronyl-nitroxide radical versus their baseline conductance (parameter d). The normalized height is defined as the difference between the conductance at zero-bias and the baseline conductance (parameter d), divided by the baseline conductance.

References

(1) Sato, S.; Yoshimasa, Y.; Fujita, D.; Yagi-Utsumi, M.; Yamaguchi, T.; Kato, K.; Fujita, M. A Self-Assembled Spherical Complex Displaying a Gangliosidic Glycan Cluster Capable of Interacting with Amyloidogenic Proteins. *Angewandte Chemie International Edition* **2015**, *54*, 8435–8439.

(2) Hirel, C.; Vostrikova, K. E.; Pécaut, J.; Ovcharenko, V. I.; Rey, P. Nitronyl and Imino Nitroxides: Improvement of Ullman's Procedure and Report on a New Efficient Synthetic Route. *Chemistry* **2001**, *7*, 2007–2014.

(3) van Veen, F.; Ornago, L.; van der Zant, H. S.; El Abbassi, M. A generalized neural network approach for separation of molecular breaking traces. *Journal of Materials Chemistry C* **2023**, *11*, 15564–15570.

(4) Fano, U. Effects of Configuration Interaction on Intensities and Phase Shifts. *Physical Review* **1961**, *124*, 1866–1878.

(5) Gruber, M.; Weismann, A.; Berndt, R. The Kondo resonance line shape in scanning tunnelling spectroscopy: instrumental aspects. *Journal of Physics: Condensed Matter* **2018**, *30*, 424001.
